# Editorial: Insights in consciousness and empathy: 2022

**DOI:** 10.3389/fnbeh.2023.1298405

**Published:** 2023-10-24

**Authors:** Giuseppe Curcio, Walter Adriani

**Affiliations:** ^1^Department of Biotechnological and Applied Clinical Sciences, University of L'Aquila, L'Aquila, Italy; ^2^Center for Behavioural Sciences and Mental Health, Istituto Superiore di Sanità, Rome, Italy

**Keywords:** empathy, consciousness, theory, animal model, neural correlates

In recent years, both consciousness and empathy have been deeply investigated by several scientists interested in neurosciences. Great attention has been dedicated to different newly proposed ways to assess consciousness (Plosnić et al., 2023; Vatrano et al., 2023) as well as to the definition and description of what we mean with the term “states of consciousness” (Schoeller, 2023) and to animal models of such complex functions (Zlomuzica and Dere, 2022). The same could be said for empathy: many efforts have been undertaken to propose unified theories between neural and psycho-cognitive aspects (Maliske et al., 2023) up to the very recent challenge of artificial empathy (Christov-Moore et al., 2023), and in all cases, it was shown that our empathic behavior can change as a function of particular “environmental” features (e.g., Migliore et al., 2019).

The aim of this Research Topic is to highlight new insights, novel developments, current challenges, latest discoveries, recent advances, and future perspectives to investigate specific questions in behavioral, cognitive, and applied neuroscientific research on consciousness and empathy.

The Research Topic features some intriguing research studies and potential application to clinical populations, as well as innovative ways to use brain stimulation techniques to modulate human relationships or apply animal models to the study of such complex higher functions.

Looking at the contributions in detail, Lobbestael et al. proposed a study to explore the influence of subclinical psychopathic traits (and their three subcomponents of egocentricity, callousness, and antisociality) on the efficacy of experimentally induced self-compassion (SC) and other-compassion (OC). To this end, they administered to both student and community a questionnaire to assess the dimensional level of psychopathy. The results indicated that subclinical psychopathic traits differentially influence changes limitedly to SC, namely, a facilitation of SC can be observed after practicing compassion. The authors concluded that compassion could be a promising intervention for the wellbeing of individuals with high psychopathic and callous traits, as well as for people living with them. Moreover, with psychopathic traits linked to high levels of anger and aggression, future research is needed to evaluate the impact of compassion on these behaviors.

Moving toward a theoretical point of view, Luis et al., after a critical review of the state-of-the art of the research about the conceptualization and assessment of empathy, focused their work specifically on investigations dealing with the issue of a shared vision of empathy under both psychological and neuroscientific perspectives. This approach also included an in-depth analysis of the neural substrates and cognitive processes involved in the emphatic phenomenon and of its main theories. The output of this complex analysis was the proposal of a new theory of the self, human growth, and action (called the Inter- Processual Self theory, IPS) that, in their opinion, will be useful to go beyond what the literature has already done.

Silveira et al. investigated the aspects of emotional contagion through a mouse model, by observing neural correlates of anxiogenesis induced by living with conspecifics subjected to chronic restraint stress. More specifically, the authors investigated the activity of the anterior cingulate cortex (ACC) and the amygdala, in order to understand their role in empathy-like behaviors. After having observed that cohabitation with mice subjected to chronic restraint stress provoked anxiogenic-like behaviors, the authors showed that a local inactivation of the ACC reversed the anxiogenic-like effects induced by cohabitation, while the amygdala did not react in a specific way. Such results clearly suggest that the anxiety induced by emotional contagion is dependent on the ACC but not the amygdala and could in turn be useful to address future studies on humans, particularly in the field of psychopharmacology.

Wu et al. used a non-invasive brain stimulation technique to clarify the role of the right temporo-parietale junction (rTPJ) with respect to fair behavior in situations of advantageous and disadvantageous inequity by modulating its activation in humans. The rTPJ is acknowledged as the neural substrate of the mentalizing network, suggested as crucial in orienting human reciprocal behavior. Thus, the authors stimulated the participants with an anodal transcranial direct current stimulation (tDCS) over the rTPJ, and then asked them to play to a modified version of the *Dictator Game*. It was seen that anodal tDCS over the rTPJ increased the participants' equity choices limitedly to the disadvantageous inequity situation. Curiously, this effect was mediated by sex, so that tDCS appeared more effective in increasing female equity choices. These results show that this brain area is of key importance in inequity aversion and that its functioning can be modulated by some genetic and/or constitutional features, for example sex.

In conclusion, the papers published within the present Research Topic demonstrated that the issues of empathy and consciousness still remain a growing field of research that needs great scientific effort to deeply clarify neural correlates, show differences and similarities between animal and human models, and propose possible clinical applications.

## Author contributions

GC: Writing—review and editing. WA: Writing—review and editing.
